# Two Novel Tyrosinase (*TYR*) Gene Mutations with Pathogenic Impact on Oculocutaneous Albinism Type 1 (OCA1)

**DOI:** 10.1371/journal.pone.0106656

**Published:** 2014-09-12

**Authors:** Vadieh Ghodsinejad Kalahroudi, Behnam Kamalidehghan, Ahoura Arasteh Kani, Omid Aryani, Mahdi Tondar, Fatemeh Ahmadipour, Lip Yong Chung, Massoud Houshmand

**Affiliations:** 1 Department of Biology, Kharazmi University, Tehran, Iran; 2 Department of Medical Genetics, Special Medical Center, Tehran, Iran; 3 Department of Pharmacy, Faculty of Medicine, University of Malaya, Kuala Lumpur, Malaysia; 4 Department of Biochemistry and Molecular & Cellular Biology, Georgetown University, Washington, D.C., United States of America; 5 Department of Medical Genetics, National Institute for Genetic Engineering and Biotechnology (NIGEB), Tehran, Iran; University of Iowa, United States of America

## Abstract

Oculocutaneous albinism (OCA) is a heterogeneous group of autosomal recessive disorders resulting from mutations of the tyrosinase (*TYR*) gene and presents with either complete or partial absence of pigment in the skin, hair and eyes due to a defect in an enzyme involved in the production of melanin. In this study, mutations in the *TYR* gene of 30 unrelated Iranian OCA1 patients and 100 healthy individuals were examined using PCR-sequencing. Additionally, in order to predict the possible effects of new mutations on the structure and function of tyrosinase, these mutations were analyzed by SIFT, PolyPhen and I-Mutant 2 software. Here, two new pathogenic p.C89S and p.H180R mutations were detected in two OCA1 patients. Moreover, the R402Q and S192Y variants, which are common non-pathogenic polymorphisms, were detected in 17.5% and 35% of the patients, respectively. The outcome of this study has extended the genotypic spectrum of OCA1 patients, which paves the way for more efficient carrier detection and genetic counseling.

## Introduction

Oculocutaneous albinism (OCA) is a group of congenital heterogeneous disorders characterized by either complete or partial absence of pigment in the skin, hair and eyes because of the absence of or a defect in an enzyme involved in the production of melanin [1]. OCA's symptoms include poor visual acuity, nystagmus, iris transillumination, strabismus, photophobia, foveal hypoplasia and misrouting of the optic nerve fibers at the chiasm [2].

OCA type 1 (OCA1, MIM 203100) is the most severe form of albinism and is caused by mutations in the tyrosinase gene (*TYR*, MIM 606933; 11q14–q21). Other subtypes of OCA include OCA type 2 (OCA2, MIM#203200) caused by mutations in the OCA2 gene (15q11.2–q12), OCA type 3 (OCA3, MIM#203290) associated with mutations in the tyrosinase-related protein gene (TYRP1, 9p23) and OCA type 4 (OCA4, MIM#606574) because of mutations in the membrane-associated transporter gene (MATP, 5p13.3) [3]. The prevalence of different forms of OCA fluctuates widely in different populations [4], where OCA1 is the most common subtype found in Caucasians and accounts for about 50% of all cases worldwide [5], [6], while OCA2 is most common in Africa and accounts for about 30% of all cases worldwide [7].

There are two subtypes of OCA1: OCA1A and OCA1B. OCA1 is caused by a mutation causing a complete lack of tyrosinase activity, while mutations resulting in the retainment of some enzyme activity result in OCA1B, where some melanin pigments are accumulated over time [8]. The common features between these two forms of OCA1 are nystagmus and foveal hypoplasia with reduced visual acuity. The human tyrosinase gene (TYR, 11q14–q21, MIM 606933) has 5 exons, spans about 65 kb of the genomic DNA, and encodes a 60 kDa glycoprotein - tyrosinase type I [9]. Tyrosinase catalyzes multiple steps in melanin synthesis, including the critical first and second reactions: the hydroxylation of tyrosine to L-DOPA and the oxidation of L-DOPA to DOPA-quinone. Mutations in TYR can cause complete or partial OCA depending on residual activity [10]. Chromosome 11 contains a pseudogene known as Tyrosinase-Like Gene (*TYRL*, 11p11.2; MIM#191270). This gene shares 98.55% sequence identity with the 3′–region of the *TYR* gene, including exons 4 and 5 [11]. Tomita et al (1989) reported the first pathological mutation in the *TYR* gene [12]. Presently, the Human Gene Mutation Database (HGMD at http://www.hgmd.org/) which is the largest general mutation database, contains approximately 320 different mutations of the *TYR* gene that have been documented. In this study, the *TYR* gene was examined in individuals who met the clinical criteria proposed for OCA1, in order to characterize the associated mutations.

## Materials and Methods

### Specimen Collection and Ethical Statement

Thirty Iranian OCA1 patients, including 12 females and 18 males with a mean age of 18 years, from 30 unrelated families were clinically diagnosed between February 2009 and December 2012. All of the patients had typical features of OCA1A, as summarized in Table 1. Blood samples from these 30 OCA1 patients and 100 healthy individuals as controls were obtained from the Special Medical Center, Tehran-Iran. Written informed consent, including consent to participate in the study for genetic analysis and consent to publish, was obtained from patients, parents on behalf of children, and healthy controls, and the Medical Ethics Committee of the Special Medical Center specifically approved this study (Approval No. FF-40-3224). The exclusion criterion for the control group was any history of cancer, metabolic diseases, and nuclear and mitochondrial DNA-related diseases that may affect DNA.

**Table 1:** Clinical features of 30 Iranian OCA1 patients.

Patient	Gender	Age	Skin color	Hair color	Iris pigmentation	Nystagmus	Photophobia	Foveal Hypoplasia*
1	M	21	White	White	Hypopigmented	+	+	+
2	M	18	White	White	Hypopigmented	+	+	+
3	M	29	White	White	Hypopigmented	+	+	+
4	F	26	White	White	Hypopigmented	+	+	+
5	F	22	White	White	Hypopigmented	+	+	+
6	M	10	White	White	Hypopigmented	+	+	+
7	M	17	White	White	Hypopigmented	+	+	+
8	M	32	White	White	Hypopigmented	+	+	+
9	F	13	White	White	Hypopigmented	+	+	+
10	M	27	White	White	Hypopigmented	+	+	+
11	M	6	White	White	Hypopigmented	+	+	+
12	M	12	White	White	Hypopigmented	+	+	+
13	F	19	White	White	Hypopigmented	+	+	+
14	M	30	White	White	Hypopigmented	+	+	+
15	M	4	White	White	Hypopigmented	+	+	+
16	M	11	White	White	Hypopigmented	+	+	+
17	M	23	White	White	Hypopigmented	+	+	+
18	F	34	White	White	Hypopigmented	+	+	+
19	F	10	White	White	Hypopigmented	+	+	+
20	F	19	White	White	Hypopigmented	+	+	+
21	M	26	White	White	Hypopigmented	+	+	+
22	F	7	White	White	Hypopigmented	+	+	+
23	M	35	White	White	Hypopigmented	+	+	+
24	F	12	White	White	Hypopigmented	+	+	+
25	F	18	White	White	Hypopigmented	+	+	+
26	M	5	White	White	Hypopigmented	+	+	+
27	M	28	White	White	Hypopigmented	+	+	+
28	F	3	White	White	Hypopigmented	+	+	+
29	F	11	White	White	Hypopigmented	+	+	+
30	M	14	White	White	Hypopigmented	+	+	+

*It is based on fundus exam.

### DNA extraction and Polymerase Chain Reaction (PCR)

**Figure 1:**
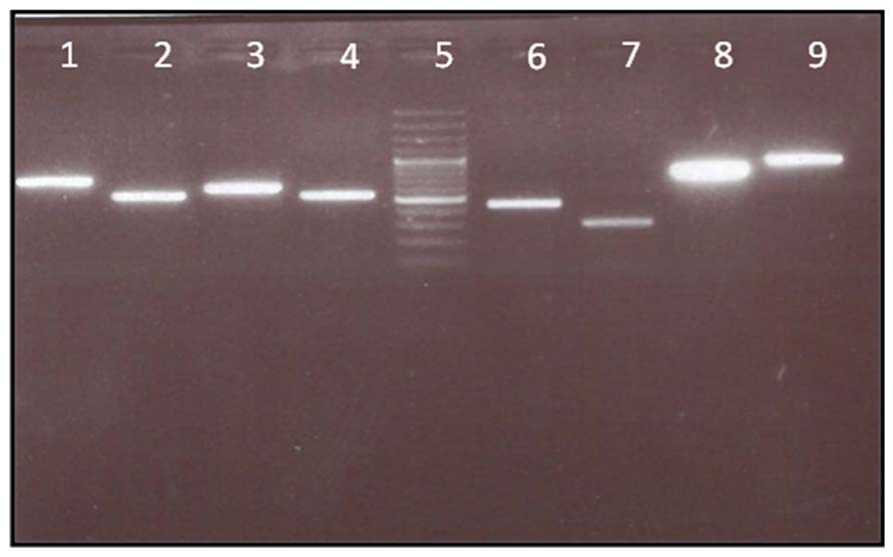
Agarose gel electrophoresis of PCR product. The PCR products electrophoresed on a 1.5% agarose gel. From left: lane 1: exon 1–1 (700 bp), lane 2: exon 1–2 (500 bp), lane 3: exon 1–3 (600 bp), lane 4: exon 1–4 (500 bp), Lane 5: DNA ladder (Thermo Scientific Gene Ruler 100 bp #SM0241/2/3), Lane 6: Exon 2 (450 bp), Lane 7: Exon 3 (300 bp), Lane 8 Exon 4(790 bp), Lane 9: Exon5 (920 bp).

Genomic DNA was extracted from blood samples of each individual, using a QIAmapDNA micro Kit (QIAGEN#56304). The exons 1 to 3 PCR primers for amplification of the *TYR* gene are shown in Table 2. Briefly, PCR was performed in a 25 µl reaction volume containing 50–100 ng of genomic DNA, 2.5 µl of 10×PCR buffer, 0.1 mM of each dNTP, 1 mM of MgCl2, 0.1 µM of each primer, and 0.5 units of *Taq* polymerase (CinnaGen, Iran) in a thermocycler (Eppendorf, Humburg). For all amplicons, the genomic DNA was denatured at 94°C for 5 min, followed by 35 cycles of denaturation at 94°C for 1 min. The annealing temperature differed according to the Tm (°C) value of each primer set (Table 2). The extension was at 72°C for 1 min and the final extension was at 72°C for 10 min. In addition, to avoid co-amplification of the pseudogene *TYRL*, the Chaki PCR protocol [11] was applied for exons 4 and 5. The PCR products were examined for specificity via 1.5% agarose gel electrophoresis (Figure 1).

**Table 2:** PCR Primer pairs for amplification of the *TYR* gene in OCA1 patients.

Gene name	Primer name	Exon	Locus (nDNA)	Primer sequence (5′ to 3′)	Amplicon size (bp)	Temperature (°C)
TYR	AL-1-1F	1–1*	88910556	CAAACTGAAATTCAATAACATATAAGG	678	59
TYR	AL-1-1R		88911233	GTGGACAGCATTCCTTCTCC		
TYR	AL-1-2F	1–2*	88910948	TTCAGAGGATGAAAGCTTAAGATAAA	521	59
TYR	AL-1-2R		88911468	CGTCTCTCTGTGCAGTTTGG		
TYR	AL-1-3F	1–3*	88911171	CTGGCCATTTCCCTAGAGC	605	60
TYR	AL-1-3R		88911775	CCACCGCAACAAGAAGAGTC		
TYR	AL-1-4F	1–4*	88911487	CATCTTCGATTTGAGTGCCC	521	59
TYR	AL-1-4R		88912007	CCCTGCCTGAAGAAGTGATT		
TYR	AL-2F	2	88924298	CCAACATTTCTGCCTTCTCC	442	60
TYR	AL-2R		88924739	TCAGCTAGGGTCATTGTCGAT		
TYR	AL-3F	3	88960909	AGTTATAAATCAAATGGGATAATCA	296	56
TYR	AL-3R		88961204	ACATTTGATAGGCACCCTCT		
TYR	AL-4F	4	89017552	CTGTTTCCAATTTAGTTTTATAC	790	56
TYR	AL-4R		89018341	TACAAAATGGCCTATGTTAAGC		
TYR	AL-5F	5	89028218	TGTCTACTCCAAAGGACTGT	924	55
TYR	AL-5R		89029138	GGCACTTAGCTGGATGTGTT		

*Shows that due to the large size of exon 1, it is divided into four overlapping fragments.

### DNA sequencing and in silico analysis of the variants

The PCR products were sequenced with the forward or reversed primers on an ABI 3700 sequencer (Kosar Company, Tehran) and compared with the wild-type *TYR* sequence (NM 000372.3) at the NCBI Reference Sequence Database (http://www.ncbi.nlm.nih.gov/refseq/), using the FinchTV program. Identification of the mutations at protein level was verified via the Human Gene Mutation Database (HGMD). For the novel mutations that were found in patients, further molecular tests were performed on the DNA of their parents and the 100 healthy individuals. To predict the functional effects of novel mutations, the sequence alterations were assessed by the in silico prediction algorithms SIFT [13], Polyphen-2 [14], and I-Mutant 2.0 (http://folding.biofold.org/i-mutant/i-mutant2.0.html).

### Statistical analysis

Fisher's exact test using SPSS (Statistical Package for the Social Sciences, version: 13) was used to analyze the relationship between the presence of novel mutations in patients and control groups; where *p*-values <0.05 were regarded as statistically significant.

## Result

**Figure 2:**
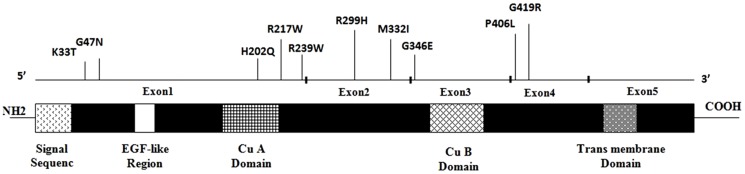
Reported mutations in this study are distributed on the *TYR* gene.

In our study, the clinical diagnosis of 30 Iranian OCA1A patients was confirmed as through molecular screening of mutations in the TYR gene in 19 patients (Table 3). Twelve different *TYR* missense mutations were identified, where two of them have not been previously reported (Figure 2, Table 4).*TYR* mutations were homozygous in 18 of the cases. These mutations were observed in the compound heterozygous state in one patient (Patient 19). Additionally, patients 3, 9, 12, 13, 18 and 24 were heterozygous for these mutations (Table 3).

**Table 3:** *TYR* mutations and polymorphisms in 30 Iranian patients.

Patient No.	Mutation 1	Mutation 2	Polymorphisms	Molecular diagnosis
1	**c.265T>A (C89S)** *	**c.265T>A (C89S)**	-	OCA1
2	-	-	p.R402Q(Hetero)	-
3	c.606T>G (H202Q)	-	p.S192Y(Hetero)	-
4	c.1217C>T(P406L)	c.1217C>T(P406L)	-	OCA1
5	c.1217C>T(P406L)	c.1217C>T(P406L)	-	OCA1
6	c.1255G>A(G419R)	c.1255G>A(G419R)	-	OCA1
7	c.606T>G (H202Q)	c.606T>G (H202Q)	-	OCA1
8	c.896G>A(R299H)	c.896G>A(R299H)	-	OCA1
9	c.1217C>T(P406L)	-	p.S192Y(Hetero)	-
10	c.649C>T (R217W)	c.649C>T (R217W)	-	OCA1
11	-	-	p.S192Y(Homo)	-
12	c.1037G>A(G346E)	-	p.R402Q(Hetero)	-
13	c.98A>C(K33T)	-	p.R402Q(Hetero)	-
14	c.1255G>A(G419R)	c.1255G>A(G419R)	-	OCA1
15	-	-	p.R402Q(Hetero)	-
16	c.996G>A(M332I)	c.996G>A(M332I)	-	OCA1
17	c.715C>T(R239W)	c.715C>T(R239W)	-	OCA1
18	c.606T>G (H202Q)	-	p.S192Y(Hetero)	-
19	**c.539A>G (H180R)**	c.1037G>A(G346E)	-	OCA1
20	c.140G>A (G47D)	c.140G>A (G47D)	-	OCA1
21	c.1037G>A(G346E)	c.1037G>A(G346E)	-	OCA1
22	c.715C>T(R239W)	c.715C>T(R239W)	-	OCA1
23	c.649C>T (R217W)	c.649C>T (R217W)	-	OCA1
24	c.1217C>T(P406L)	-	p.S192Y(Hetero)	-
25	c.140G>A (G47D)	c.140G>A (G47D)	-	OCA1
26	c.715C>T(R239W)	c.715C>T(R239W)	-	OCA1
27	c.98A>C(K33T)	c.98A>C(K33T)	-	OCA1
28	c.896G>A(R299H)	c.896G>A(R299H)	-	OCA1
29	-	-	p.R402Q(Hetero)	-
30	-	-	p.R402Q(Hetero)	-

*Hetero: Heterozygous; Homo: Homozygous; (-): undetected; New mutations are in bold.

**Table 4:** The identified mutations in the *TYR* gene from OCA1 patients in our study.

Nucleotide change	Amino acid change	Location	Frequency (%)	Status	Reference
c.98A>C	K33T	Exon 1	2(5.4)	Homo (1)*; Hetero (1)	Reported [6]
c.140G>A	G47D	Exon 1	2(5.4)	Homo (2)	Reported [42]
**c.265T>A**	**C89S**	**Exon 1**	**1(2.7)**	**Homo (1)**	**Not reported (New)**
c.575C>A	S192Y	Exon 1	5(13.5)	Homo (1); Hetero (4)	Reported [43]
**c.539A>G**	**H180R**	**Exon 1**	**1(2.7)**	**Hetero (1)**	**Not reported (New)**
c.606T>G	H202Q	Exon 1	3(8.1)	Homo (1); Hetero (2)	Reported [44]
c.649C>T	R217W	Exon 1	2(5.4)	Homo (2)	Reported [45]
c.715C>T	R239W	Exon 1	3(8.1)	Homo (3)	Reported [46]
c.896G>A	R299H	Exon 2	2(5.4)	Homo (2)	Reported [45]
c.996G>A	M332I	Exon 2	1(2.7)	Homo (1)	Reported [47]
c.1037G>A	G346E	Exon 3	3(8.1)	Homo (1); Hetero (2)	Reported [48]
c.1205G>A	R402Q	Exon 4	6(16.2)	Hetero (6)	Reported [24]
c.1217C>T	P406L	Exon 4	4(10.8)	Homo (2); Hetero (2)	Reported [49]
c.1255G>A	G419R	Exon 4	2(5.4)	Homo (2)	Reported [16]

*The number in parenthesis in the status column shows the number of patients; New mutations are in bold.

**Figure 3:**
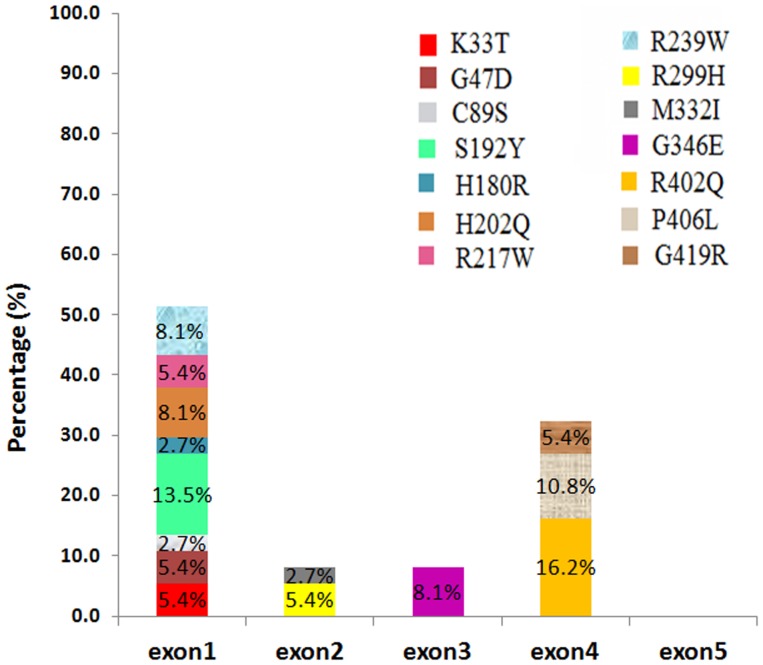
Distribution of mutations on the *TYR* gene in OCA1 patients. Bar diagram indicates the percentage of mutations that were found in this study.

**Figure 4:**
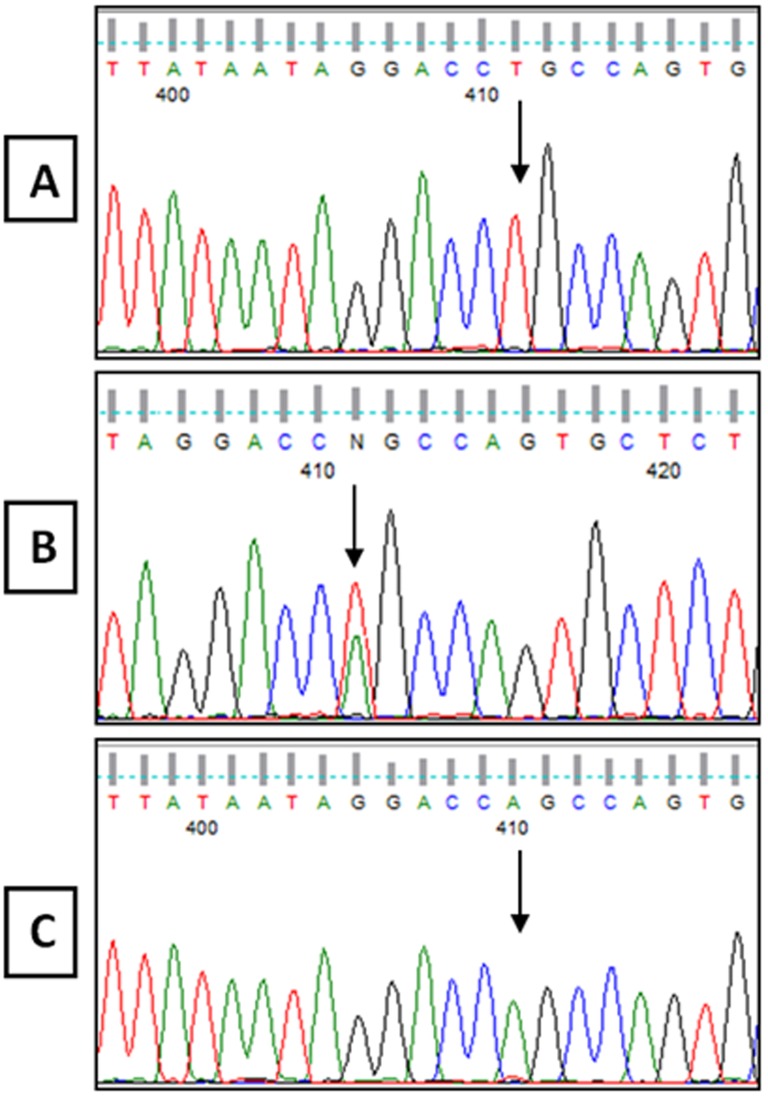
DNA sequencing result from Exon 1 of the *TYR* gene, showing c.265 T>A mutation. **A**: Normal Sequence from control. **B**: Sequence from unaffected parents showing the c.265 T>A heterozygous mutation. **C**: Sequence from patient 1 with a new c.265 T>A homozygous mutation.

**Figure 5:**
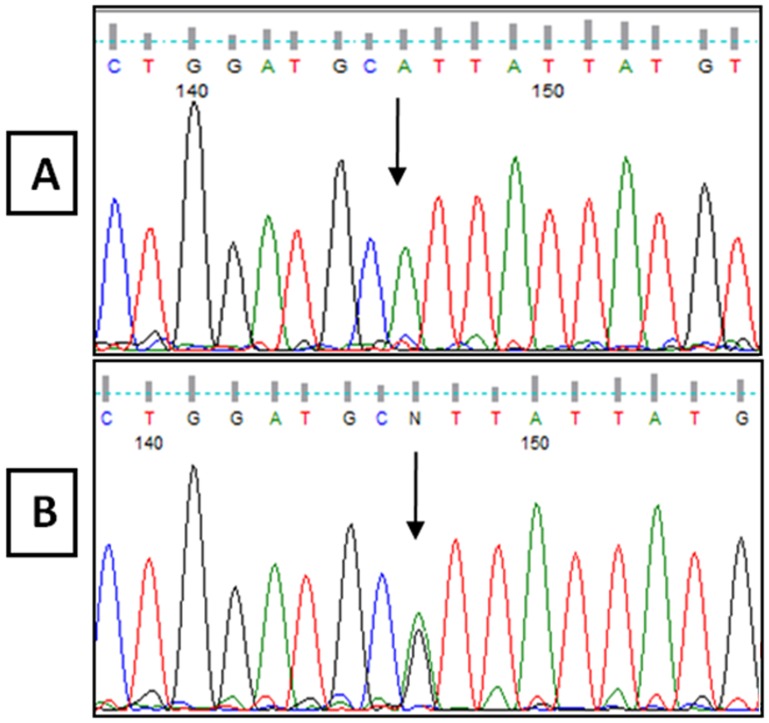
DNA sequencing result from Exon 1 of the *TYR* gene showing c.539 A>G mutation. **A**: Normal Sequence from control and unaffected father. **B**: Sequence from patient 19 and an unaffected mother showing the c.539 A>G heterozygous mutation.

**Figure 6:**
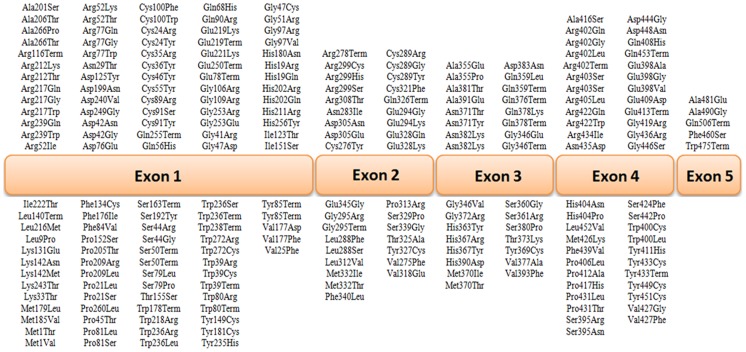
Database of tyrosinase (*TYR*) gene mutations. [50] The mutations identified in OCA1 for each exon are described in the upper and lower half of the schema.

The R402Q and S192Y variants, which are common non-pathogenic polymorphisms, were detected in 6 and 5 cases, respectively (Figure 3 and Table 3). In patient 1, a new homozygous c.265T>A change in codon 89, which resulted in a cysteine to serine conversion (C89S), was identified (Figure 4). Moreover, patient 19 was compound heterozygous for the reported G346Q and new H180R (c.539A>G) mutations (Figure 5). The new p.C89S and p.H180R mutations were significantly (*p*<0.05) detected in the patients (Table 5), and compared to global database of *TYR* gene mutations (Figure 6).To predict the possible effects of these new mutations on the structure and function of tyrosinase, the mutations were analyzed using SIFT, PolyPhen and I-Mutant 2 softwares (Table 5). Here, SIFT results indicated that both C89S and H180R mutations were predicted as deleterious, with SIFT scores of −9.497 and −7.772, respectively. Using the I-Mutant server, prediction based on the sign of the free energy change value (sign of DDG) for the new C89S and H180R mutations showed that these mutations decrease the stability of the protein. Based on the PolyPhen score, both the C89S and H180R mutations were found as “Probably Damaging” to protein structure and function, with a score of 1.000, although further research is required to confirm these in silico findings.

**Table 5:** Statistical and Bioinformatics Analysis of two novel pathogenic mutations.

Novel mutation	Statistical analysis	Bioinformatic analysis				
	*p*-value	Polyphen 2		SIFT		I-Mutant 2.0
		Prediction	Score	Prediction	Score	Prediction (sign of DDG)
C89S	0.048	Probably Damaging	1	Deleterious	−9.497	decrease stability
H180R	0.048	Probably Damaging	1	Deleterious	−7.772	decrease stability

Fisher's exact test using the SPSS was used for statistical analysis of novel mutations. A *p*-value of <0.05 is considered statistically significant. Novel mutations were analyzed by three computational methods PolyPhen 2 (benign/damaging), SIFT (tolerated/deleterious), and I-Mutant 2.0 (increase stability/decrease stability) for Bioinformatics analysis in order to predict the functional impact of novel amino acid changes.

*p*-value: statistically significant (*p*<0.05).

PolyPhen Prediction Score: benign ≤0.5; probably damaging (0.5<).

SIFT Prediction Score: deleterious (≤0.05); tolerated (≥0.05).

I-Mutant 2.0 Prediction: sign of DDG: decrease stability or increase stability.

## Discussion

Missense mutation is the most common mutational type that is correlated to OCA1 [15]. For the first time, King et al [16] recognized that most of the *TYR* missense mutations were located in four areas of the gene. Until now, *TYR* missense mutations are delineated by five key functional sites of the enzyme. Two locations are the copper-binding sites. Others are located at the 3′-end of the copper B-binding region near the amino terminus of the protein, and between the CuA and CuB domains. According to many reports, mutations in the Cu-binding site change the conformation of the a-helical regions [9] as well as the position of the histidine residues, which either could prevent proper binding of Cu to the histidine ligands or prevent TYR-Cu interaction.

In this study, the missense mutations were detected in all exons of the *TYR*, except for exon 5, where 51.3% and 32.4% of the mutations were found in exon 1 and exon 4, respectively, which could be considered as mutation hot spots of the *TYR* gene. Fukai *et al* (1995) reported that the R402Q mutation was associated with OCA1 incidence. At the physiological temperature (37°C), the p.R402Q tyrosinase has a reduced activity and is retained in the endoplasmic reticulum of melanocytes [17]–[20]. The contribution of the p.R402Q temperature-sensitive variant to the albino phenotype has been heavily debated in literature [10]. By itself this variant is not sufficient to cause albinism [21], [22]. It is possible that p.R402Q causes partial albinism only when paired with certain a genetic background [23], [24]. In support of this, several observers have noted that the p.R402Q variant is more common in OCA patients with one *TYR* mutation than in patients with two mutations [10]. However, in our study, of 6 patients (16.2%) with the heterozygous p.R402Q variant, two (Patients 12 and13) were heterozygous for one reported missense mutation in the *TYR* gene, G346E and K33T, while no mutation was found for the remaining 4 patients (Table 3).

The p.S192Y variant, which was reported as an SNP (rs1042602) in many populations such as Caucasians, Africans, Japanese and South Asians [25], was detected in 5 patients in our study, where 4 were heterozygous for one reported missense mutation, and one was only homozygous for the p.S192Y variant (Table 3). According to our results, the new C89S (c.265T>A) mutation was not found in the 100 control individuals. Moreover, further analysis of the parents with the normal phenotype indicated that they were heterozygous for this mutation (Figure 4). Additionally, the c.265 T>C mutation in this codon, which causes a cysteine to arginine substitution, was previously reported as a pathogenic mutation by Spritz [26]. The region spanning codons 73–107 is entirely conserved between human [27], [28] and mouse [29], [30] tyrosinases, as this region is one of the six potential N-glycosylation sites (codons 86–88), it could be vital for the function of tyrosinase [26]. Furthermore, cysteine residues in tyrosinase play an important role in the proper folding and maintenance of the tyrosinase tertiary structure in humans [31]. Alterations of these cysteine residues, such as C89S mutations in exon 1 could inactivate the protein and cause OCA1 [3]. Therefore, C89S could be considered as a pathogenic mutation.

According to our study, the new H180R (c.539A>G) mutation has not been previously reported, while in this codon (180), another pathogenic mutation (c.538C>A) that converts a His residue to an Asn residue (H180N) was previously reported. In addition, the H180R mutation is located in a sensitive domain of the enzyme, which converts one of the His residues that are bound to CU ion (metal binding site) [32]. Moreover, the father and mother of this patient were heterozygous for the G346Q and H180R mutations, respectively, and showed a normal phenotype (Figure 5). Therefore, the H180R could be considered as a pathogenic mutation.

In this study, 5 patients did not show any causative mutations in the *TYR* gene, which is probably due to the involvement of other OCA genes, such as the OCA2 gene, undiscovered OCA genes [33], [34], variants in the promoter or other regulatory elements which were not detected by DNA sequencing [35], synergistic or epistatic heterozygosity among known genes [36], [37], dominant mutations which are not detected as pathogenic because of ethnic/pigmentation background [23], [38], undetected splicing mutations [22], [39], undetected large deletions which are not recognized by DNA sequencing [6], [40], and undetected coding mutations because of allele dropout in sequencing [41].

In summary, we identified ten previously-reported missense mutations and two novel pathogenic mutations, c.265T>A (C89S) and c.539A>G (H180R), in Iranian patients with OCA1. However, further research is required to distinguish the subtype of OCA1 in these patients and to determine the biological role of these mutations that may affect tyrosinase enzymatic activity. In conclusion, the outcome of this study has extended the genotype spectrum of Iranian patients with pathogenic impact on oculocutaneous albinism type 1, which paves the way for a more efficient diagnosis and genetics counseling for carrier detection with this disorder in Iran.
